# The Effect of Monthly Sulfadoxine-Pyrimethamine, Alone or with Azithromycin, on PCR-Diagnosed Malaria at Delivery: A Randomized Controlled Trial

**DOI:** 10.1371/journal.pone.0041123

**Published:** 2012-07-19

**Authors:** Mari Luntamo, Anne-Maria Rantala, Steven R. Meshnick, Yin Bun Cheung, Teija Kulmala, Kenneth Maleta, Per Ashorn

**Affiliations:** 1 Department of International Health, University of Tampere Medical School, Tampere, Finland; 2 Department of Epidemiology, Gillings School of Global Public Health, University of North Carolina, Chapel Hill, North Carolina, United States of America; 3 Singapore Clinical Research Institute, Singapore, Singapore; 4 Duke-NUS Graduate Medical School Singapore, Singapore; 5 College of Medicine, University of Malawi, Blantyre, Malawi; 6 Department of Pediatrics, Tampere University Hospital, Tampere, Finland; London School of Hygiene and Tropical Medicine, United Kingdom

## Abstract

**Background:**

New regimens for intermittent preventive treatment in pregnancy (IPTp) against malaria are needed as the effectiveness of the standard two-dose sulfadoxine-pyrimethamine (SP) regimen is under threat. Previous trials have shown that IPTp with monthly SP benefits HIV-positive primi- and secundigravidae, but there is no conclusive evidence of the possible benefits of this regimen to HIV-negative women, or to a population comprising of both HIV-positive and –negative women of different gravidities.

**Methods:**

This study analyzed 484 samples collected at delivery as part of a randomized, partially placebo controlled clinical trial, conducted in rural Malawi between 2003 and 2007. The study included pregnant women regardless of their gravidity or HIV-infection status. The participants received SP twice (controls), monthly SP, or monthly SP and two doses of azithromycin (AZI-SP). The main outcome was the prevalence of peripheral *Plasmodium falciparum* malaria at delivery diagnosed with a real-time polymerase chain reaction (PCR) assay.

**Findings:**

Overall prevalence of PCR-diagnosed peripheral *P. falciparum* malaria at delivery was 10.5%. Compared with the controls, participants in the monthly SP group had a risk ratio (95% CI) of 0.33 (0.17 to 0.64, P<0.001) and those in the AZI-SP group 0.23 (0.11 to 0.48, P<0.001) for malaria at delivery. When only HIV-negative participants were analyzed, the corresponding figures were 0.26 (0.12 to 0.57, P<0.001) for women in the monthly SP group, and 0.24 (0.11 to 0.53, P<0.001) for those in the AZI-SP group.

**Conclusions:**

Our results suggest that increasing the frequency of SP administration during pregnancy improves the efficacy against malaria at delivery among HIV-negative women, as well as a population consisting of both HIV-positive and –negative pregnant women of all gravidities, in a setting of relatively low but holoendemic malaria transmission, frequent use of bed nets and high SP resistance.

**Trial Registration:**

ClinicalTrials.gov NCT00131235

## Introduction

Malaria is one of the most important preventable causes of poor maternal health and adverse birth outcomes [1]. In sub-Saharan Africa (SSA), an estimated 25 million pregnant women are in danger of *Plasmodium falciparum* infections every year [2]. Fortunately, much of the malaria-associated morbidity and mortality can be prevented by intermittent preventive treatment in pregnancy (IPTp), and 35 of 45 sub-Saharan African countries had adopted IPTp with sulfadoxine-pyrimethamine (SP) as a national policy by the end of 2010 [3]. However, the effectiveness of the standard two-dose SP IPTp is under threat due to increasing SP-resistance, declined malaria immunity among women infected with human immunodeficiency virus (HIV), possibly too long treatment interval, and difficulties in practical implementation [4]–[13]. Thus, new antimalarial regimens for IPTp are needed, but finding an alternative to SP has proven difficult. Therefore the WHO continues to recommend the use of SP IPTp for pregnant women at risk of *P. falciparum* in SSA [3]. Increasing the dosing frequency of SP has been considered as one possibility to improve the effectiveness of SP IPTp while alternative regimens are being explored.

At least four trials have tested the efficacy of an IPTp regimen that contains monthly SP-dosing [14]–[17]. However, three of these trials focused on defined risk groups and not an unselected population of pregnant women, which typically comprises the actual target group for IPTp [14]–[16]. A Kenyan and a Malawian study enrolled participants in their first or second pregnancy [14], [15], and a Zambian sample included only HIV-positive women [16]. These three trials, in aggregate, suggest that monthly SP results in less placental and peripheral maternal malaria at delivery and a higher birth weight among HIV-positive primi- and secundigravidae [13]. However, there is no conclusive evidence of the benefits of monthly SP for HIV-negative women, for HIV-positive multigravidae, or for a population comprising of both HIV-positive and –negative women of different gravidities.

In our previously reported trial from Malawi we included pregnant women regardless of their gravidity or HIV-infection status [17]. A total of 1320 participants were randomized to receive either two doses of SP (control), monthly SP, or a combination of monthly SP and two doses of azithromycin (AZI-SP). Compared with the controls, the trial documented a significantly lower incidence of both preterm delivery and low birth weight (LBW) in the AZI-SP, but not in the monthly SP group. Peripheral malaria prevalence assessed by conventional microscopy was higher among controls (approximately 5%) than among women in both intervention groups (approximately 2%), both around 32 gestational weeks and at delivery. However, these intergroup differences were statistically significant only around 32 gestational weeks – possibly because microscopic malaria samples at delivery were available only for 481 individuals who gave birth at a health facility. Hence, we could not establish if the trial regimens reduced malaria exposure in late pregnancy, nor whether the differences in pregnancy outcomes between the monthly SP and AZI-SP groups were due to an additional antimalarial effect of azithromycin or other factors.

In a subsequent study, we applied a polymerase chain reaction (PCR) – based method to diagnose malaria from peripheral blood samples collected at delivery in the above described trial. With this more sensitive method, we documented an almost five fold higher prevalence of *P. falciparum* parasitaemia than earlier diagnosed with microscopy [18]. In the current study we used the results of this PCR-methodology to assess the effect of monthly SP and AZI-SP treatments on peripheral malaria parasitaemia at delivery in a population that consisted of both HIV-positive and –negative participants of all gravidities.

## Materials and Methods

### Study Design and Outcomes

This study is a secondary analysis of peripheral blood samples collected at delivery for real-time PCR assay targeting *P. falciparum* as part of the Lungwena Antenatal Intervention Study (LAIS), which has been described in detail elsewhere [17]. Of the 1320 LAIS participants, these blood samples were collected only from the women who delivered at a local health facility. The LAIS enrolled both HIV-positive and –negative pregnant women of all gravidities into a randomized, partially placebo controlled clinical trial, which was conducted in rural Malawi between 2003 and 2007. The study hypothesis was that preterm delivery and other adverse pregnancy outcomes could be reduced, and maternal health improved by intermittent preventive treatment (IPT) of pregnant women with monthly sulfadoxine-pyrimethamine, alone or in combination with two doses of azithromycin. The protocol for LAIS and the CONSORT checklist for this paper are available as supporting information; see Protocol S1 and Checklist S1.

### Ethics Statement

The trial was performed according to Good Clinical Practice guidelines and the ethical standards of Helsinki Declaration. The protocol was approved by the College of Medicine Research and Ethics Committee, University of Malawi, Malawi and the Ethical Committee of Pirkanmaa Hospital District, Finland. Key details of the protocol were published at the clinical trial registry of the National Library of Medicine, Bethesda, Md, USA (http://www.clinicaltrials.gov, trial identification NCT00131235). Only participants who signed or thumb-printed an informed consent form were enrolled in the study.

### Study Site and Participants

The target population of LAIS comprised pregnant women who came between December 2003 and October 2006 for antenatal care to Lungwena Health Centre, Mangochi district, southern Malawi. Malaria is holoendemic at this rural site [19]. Inclusion criteria for the study were gestational age 14–26 weeks by ultrasound assessment, felt movements of the fetus, availability for follow-up, and informed consent. Exclusion criteria included severe illness, receipt of azithromycin during the current pregnancy or SP within preceding 28 days, allergy to study drugs, and any previous serious allergic reaction. In the analyses presented in the current paper only participants with available real-time PCR results for *P. falciparum* malaria at delivery were included.

### Study Interventions and other Medication

Participants in the control group (“control”) received standard Malawian antenatal care including IPTp with SP (three tablets orally, each containing 500 mg sulfadoxine and 25 mg pyrimethamine), and a placebo to azithromycin, both given twice during pregnancy: at enrollment and between 28^th^ and 34^th^ weeks of gestation. Participants in the first intervention group (“monthly SP”) received otherwise the same treatment, but SP was given monthly from enrollment until 37 gestational weeks. Participants in the second intervention group (“AZI-SP”) received otherwise the same treatment as the monthly SP group, but instead of placebo, they received active azithromycin twice (two tablets orally, each containing 500 mg of azithromycin): at enrollment visit and between 28^th^ and 34^th^ weeks of gestation. All participants received ferrous sulphate (200 mg/day) and folic acid (0.25 mg/day) throughout pregnancy.

Participants diagnosed with malaria at enrollment or at 28–34^th^ week visit received the normal pre-packed study drugs which included SP for all study groups. Laboratory-confirmed malaria at any other point was treated with quinine (two tablets orally three times a day for seven days, each containing 300 mg of quinine).

SP tablets were purchased from Malawi Central Medical Stores that were supplied by Pharmanova, Ipca Laboratories Ltd, F.Hoffmann-La Roche Ltd, and Universal Corporation Kenya Ltd. Both active azithromycin and its placebo were manufactured and donated by Pfizer Inc. We did not perform any pharmacological tests on the study drugs.

### Randomization and Enrollment

A researcher not involved in data collection generated a randomization code list in blocks of nine. Based on this list, an individual drug box was pre-packed for each identification number. The drug box contained appropriate study drugs for each planned study visit in separate opaque drug envelopes labeled with identification number and visit information. Individual slips containing unique identification numbers, but not group allocation, were sealed in individual opaque randomization envelopes. The envelopes were grouped into randomization blocks to ensure similar allocation rates to each group.

At enrollment visit, the research personnel interviewed individuals interested in participating in the study about their socioeconomic status, obstetric history and bed net use, gave pre-test HIV-counseling, and performed an antenatal and laboratory examinations. Testing for HIV was optional. Eligible individuals signed or thumb-printed informed consent and picked one randomization envelope which contained an identification number. A research assistant not involved in outcome assessment gave the corresponding pre-packed study drugs to the participant under direct observation.

### Follow-up

At follow-up visits (at four-week intervals until 36 completed gestational weeks and weekly thereafter) the research personnel conducted an antenatal examination. The participants were offered post-test HIV counseling and prevention of mother to child transmission. At the visit during 28th–34th weeks of gestation, a malaria test was conducted. At each visit, the participant took the appropriate pre-packed study drugs under direct observation.

Upon notification of a delivery, a research assistant visited the delivery site. She collected data on delivery, examined the newborn and gave her/him nevirapine or placebo based on maternal HIV status. From participants who delivered at a local health facility, maternal peripheral venous blood was collected as thin and thick blood films, and as dried blood spots on filter paper.

### Laboratory Procedures

Peripheral blood *P. falciparum* parasitaemia at delivery was diagnosed with a *P. falciparum* lactate dehydrogenase-specific (pfldh) real-time PCR assay using DNA extracted from dried blood spots, as previously described [18].

### Statistical Methods

Statistical analysis was carried out with Stata 9.2 (StataCorp, College Station, USA). We estimated risk ratio and risk difference for comparison of binary end-points at a single time point. To prevent inflated type I errors due to multiple comparisons, we began hypothesis testing with global null hypotheses of all three groups being identical before doing pair-wise comparisons. We tested the hypotheses with Fisher’s exact test.

The proportion of women with no previous pregnancies was higher in the control and monthly SP groups than in the AZI-SP group, and the proportion with peripheral malaria parasitaemia at enrollment was higher in the control group than in the intervention groups. As sensitivity analyses, we adjusted for these two covariates as categorical variables by generalized linear models and compared the adjusted and main analyses. We performed tests for interaction between the interventions and HIV status (those whose status was known), the number of previous pregnancies (classified as none, and one or more), and bed net use using the likelihood ratio test, and did analyses stratified by the same variables.

## Results

### Participants and Received Treatment

**Figure 1:**
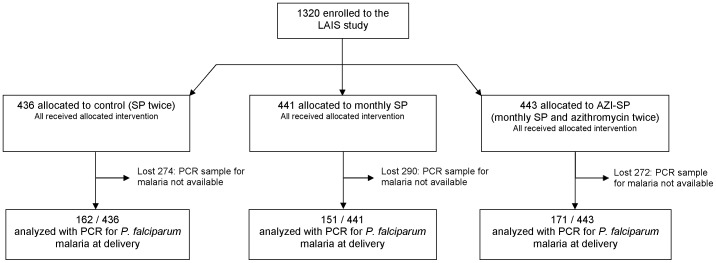
Participant flow in CONSORT recommended format.

Of the 1320 LAIS participants [17], 484 (36.7%) had dried blood spots taken at delivery at a local health facility for the real-time PCR assay targeting *P. falciparum*, and they formed the study population (figure 1). They were on average younger, had more education, were more often primigravida, made more health centre visits, and received slightly more study drugs than the 836 (63.3%) participants excluded from the current analysis due to missing PCR-results (table 1). Except for the number of previous pregnancies and the prevalence of microscopic malaria parasitaemia (32.7%, 34.4%, 19.9% were primigravida, and 14.8%, 8.6%, 3.5% had baseline malaria in the in the control, monthly SP and AZI-SP groups, respectively), the characteristics of the three study groups were comparable at enrollment.

The mean (standard deviation, SD) number of scheduled SP treatments received was 2.0 (0.2) in the control, 4.2 (0.9) in the monthly SP, and 4.1 (0.8) in the AZI-SP group. Women in the AZI-SP group received a mean (SD) of 2.0 (0.1) azithromycin doses. The mean number (SD) of quinine doses given at non-scheduled outpatient visits was 0.04 (0.23) for the control, 0.05 (0.22) for the monthly SP, and 0.02 (0.15) for the AZI-SP group (P = 0.415). Against the trial protocol some SP-doses were given also at the non-scheduled visits, but the number of these was very small and there were no differences between the groups (4 participants each received one additional dose: 1 in control, 2 in monthly SP and 1 in AZI-SP group).

**Table 1:** Baseline characteristics at enrollment.

Characteristic	Sub-characteristic	Control (SP twice)N = 162	Monthly SPN = 151	AZI-SPN = 171	LAIS participants not included in the current analysis (all groups combined)N = 836
Mean (SD) age in years		24 (7)	24 (6)	25 (6)	25 (7)
Number (%) of literate participants		49 (30.3%)	47 (31.1%)	65 (38.0%)	223 (26.7%)
Mean (SD) years of schooling completed		2.5 (2.9)	2.4 (2.9)	2.8 (2.9)	2.0 (2.6)
Mean (SD) height in cm		154.8 (5.9)	154.4 (5.2)	155.0 (5.5)	155.2 (5.5)
Mean (SD) BMI as kg/m^2^		21.7 (2.1)	21.8 (2.3)	22.1 (2.1)	21.7 (2.2)
Mean (SD) gestational age at enrollment in weeks		20.3 (3.0)	19.5 (3.2)	19.9 (3.0)	20.2 (3.0)
Number (%) of previous pregnancies					
	None	53 (32.7%)	52 (34.4%)	34 (19.9%)	167 (20.0%)
	One or more	109 (67.3%)	99 (65.6%)	137 (80.1%)	669 (80.0%)
Number (%) of women owning a bed net		123 (75.9%)	107 (70.9%)	130 (76.0%)	608 (72.7%)
Number (%) of women who used bed net the night before enrollment		104 (64.2%)	88 (58.3%)	100 (58.5%)	505 (60.4%)
Maternal HIV status, number (%)					
	Positive	18 (11.1%)	19 (12.6%)	23 (13.5%)	101 (12.1%)
	Negative	129 (79.6%)	119 (78.8%)	129 (75.4%)	656 (78.5%)
	Unknown	15 (9.3%)	13 (8.6%)	19 (11.1%)	79 (9.5%)
Number (%) with microscopic peripheral blood malaria parasitaemia at enrollment		24 (14.8%)	13 (8.6%)	6 (3.5%)	74* (8.9%)
Mean (SD) blood Hb concentration as g/L		109 (18)	110 (16)	110 (20)	111 (19)

Abbreviations: AZI-SP, intervention group that received monthly SP and two doses of azithromycin; SD, standard deviation; BMI, body mass index; Hb, hemoglobin.

* Denominator is 835 because of one missing malaria result.

### Study Outcome

The overall prevalence of PCR-diagnosed peripheral *P. falciparum* malaria at delivery was 10.5%, with significant differences between women in the control group and those who received monthly SP either alone or in combination with azithromycin (P<0.001, table 2). Compared with the controls, participants in the monthly SP group had a risk ratio (95% confidence interval, CI) of 0.33 (0.17 to 0.64, P<0.001) and absolute risk reduction of 13.7% (6.4 to 21.1%) for malaria at delivery. For women in the AZI-SP group, the corresponding risk ratio (RR) was 0.23 (0.11 to 0.48, P<0.001) and risk reduction 15.7% (8.7 to 22.7%).

**Table 2:** Prevalence of PCR-diagnosed peripheral malaria at delivery by study group, unadjusted and adjusted analyses.

PCR-diagnosed malaria at delivery	Number with outcome/total number with known outcome data				Comparison between monthly SP and control group		Comparison between AZI-SP and control group	
	Control	Monthly SP	AZI-SP	P-value	Risk ratio (95% CI)	P-value	Risk ratio (95% CI)	P-value
Unadjusted	33/162 (20.4%)	10/151 (6.6%)	8/171 (4.7%)	<0.001	0.33 (0.17 to 0.64)	<0.001	0.23 (0.11 to 0.48)	<0.001
Adjusted for the number of previous pregnancies					0.31 (0.16 to 0.61)	0.001	0.25 (0.12 to 0.52)	<0.001
Adjusted for microscopic malaria at enrollment					0.33 (0.17 to 0.65)	0.001	0.24 (0.11 to 0.51)	<0.001

Abbreviations: AZI-SP, intervention group that received monthly SP and two doses of azithromycin; CI, confidence interval.

### Ancillary Analyses

Sensitivity analyses, adjusting either for the number of previous pregnancies or microscopic malaria parasitaemia at enrollment, gave essentially identical results to those of the unadjusted analysis (table 2), as did an analysis adjusting simultaneously for these variables. In the latter analysis, women in the monthly SP group had a 13.7 percentage point (95% CI 6.4 to 21.1%, P<0.001) lower prevalence of PCR-positive malaria at delivery than the participants in the control group. For women in the AZI-SP group, the corresponding risk reduction was 14.5% (7.7 to 21.4%, P<0.001).

In a stratified analysis including only HIV-negative participants, those in the monthly SP group had a RR (95% CI) of 0.26 (0.12 to 0.57, P<0.001) and those in the AZI-SP group 0.24 (0.11 to 0.53, P<0.001) for PCR-diagnosed malaria at delivery, when compared with the controls (table 3). Malaria was more prevalent, and the study interventions were associated with a greater reduction in malaria prevalence, among participants who were primigravida than among those who had previous pregnancies (P = 0.009 for prevalence), and among women who did not use a bed net the night before enrollment than among those who used one (P<0.001 for prevalence). There was no significant interaction between these characteristics and the intervention on the prevalence of PCR-diagnosed malaria at delivery (P = 0.480 for HIV status at enrollment, P = 0.259 for the number of previous pregnancies, and P = 0.820 for bed net use at enrollment).

**Table 3:** Stratified analyses of PCR-diagnosed peripheral malaria at delivery by study group based on maternal HIV status, number of previous pregnancies, and maternal bed net use at enrollment.

Stratified analysis of malaria at delivery based on	Number of participants with malaria (%)					Comparison between monthly SP and control group		Comparison between AZI-SP and control group	
	All groups	Control	Monthly SP	AZI-SP	P-Value	Risk ratio (95% CI)	P-Value	Risk ratio (95% CI)	P- Value
**Maternal HIV status at enrollment**									
Positive	3/60 (5.0%)	2/18 (11.1%)	1/19 (5.3%)	0/23 (0.0%)	0.189	0.47 (0.05 to 4.78)	0.604	0	0.187
Negative	43/377 (11.4%)	29/129 (22.5%)	7/119 (5.9%)	7/129 (5.4%)	<0.001	0.26 (0.12 to 0.57)	<0.001	0.24 (0.11 to 0.53)	<0.001
**Number of previous pregnancies**									
None	23/139 (16.6%)	18/53 (34.0%)	3/52 (5.8%)	2/34 (5.9%)	<0.001	0.17 (0.05 to 0.54)	<0.001	0.17 (0.04 to 0.70)	0.003
One or more	28/345 (8.1%)	15/109 (13.8%)	7/99 (7.1%)	6/137 (4.4%)	0.028	0.51 (0.22 to 1.21)	0.175	0.32 (0.13 to 0.79)	0.011
**Maternal bed net use at enrollment**									
No	32/192 (16.7%)	21/58 (36.2%)	6/63 (9.5%)	5/71 (7.0%)	<0.001	0.26 (0.11 to 0.61)	<0.001	0.19 (0.08 to 0.48)	<0.001
Yes	19/292 (6.5%)	12/104 (11.5%)	4/88 (4.6%)	3/100 (3.0%)	0.044	0.39 (0.13 to 1.18)	0.115	0.26 (0.08 to 0.89)	0.029

Abbreviations: AZI-SP, intervention group that received monthly SP and two doses of azithromycin; CI, confidence interval.

## Discussion

The use of IPT during pregnancy can prevent malaria and the adverse outcomes it causes to pregnant women and their fetuses in SSA. In the current analysis we used a sensitive PCR–based diagnostic method to compare the antimalarial effect of the standard two-dose SP IPTp regimen with monthly SP, alone or in combination with two doses of azithromycin. Compared with the control group, the participants in the monthly SP and AZI-SP groups had relatively 67% and 77% lower prevalence of PCR-diagnosed peripheral *P. falciparum* malaria parasitaemia at delivery, respectively. In stratified analyses, HIV-negative participants of all gravidities, primigravidae regardless of their HIV-status, and women who did not use a bed net at enrollment had significantly less malaria in both intervention groups than those in the control group.

The study population was drawn from a trial which used broad inclusion criteria, random group allocation and blinding of the outcome assessors [17]. The intervention code was linked to the malaria results only after the DNA-based diagnostic tests were completed. The number of participants with available PCR-results, as well as their baseline characteristics, was similar in the three study groups, except for differences in the number of previous pregnancies and the prevalence of malaria parasitaemia. However, similar differences were seen already in the main study [17], and adjusted analyses suggested that these did not bias the results. Confidence interval calculations and hypothesis testing indicated that the probability of type I error was small. We therefore believe that the sample findings were reliable and representative, and that IPTp with monthly SP, with or without azithromycin, was indeed associated with reduced prevalence of PCR-diagnosed peripheral *P. falciparum* malaria parasitaemia at delivery in the target population from which the sample was drawn.

A secondary analysis of pooled data from three other clinical trials from Kenya, Malawi and Zambia suggested that compared with the two-dose SP regimen, the monthly regimen had a beneficial effect on malaria prevalence at delivery and birth weight among HIV-positive pregnant women in their first or second pregnancy [13]. The previous Malawian study found significantly lower peripheral malaria at delivery in the monthly SP group also among HIV-negative primi- and secundigravidae [15]. However, when this data was pooled with the data of HIV-negative participants in the Kenyan study, the analysis failed to document statistical significance [13]. None of these earlier studies addressed the efficacy of the monthly SP regimen in a representative sample that contained both HIV-negative women and those with more than one earlier pregnancy [14]–[16]. Hence, it has thus far been uncertain, whether the monthly SP regimen would best be reserved for some selected risk groups, or it should be given to all pregnant women in malaria endemic areas [13].

Our results indicate that IPTp with monthly SP can in some conditions reduce the prevalence of peripheral blood *P. falciparum* malaria parasitaemia at delivery in the latter, wider target group, as well as among HIV-negative women regardless of their gravidity. This finding is consistent with the results of a recently reported trial from Mali, which found that IPTp with three doses of SP is superior to two doses in reducing malaria prevalence at delivery, LBW and premature delivery among mainly HIV-negative women of different gravidities [12]. The sample of the current substudy was not powered to look at more direct health outcomes, but since also PCR-detectable submicroscopic parasitaemias have important consequences to maternal health and birth outcomes, the observed effect is likely to be beneficial both to the mother and the newborn [20]–[22] (Atupele Kapito-Tembo et al, unpublished results).

Besides the apparently improved efficacy against malaria, monthly dosing would solve some of the problems associated with the two-dose SP IPTp. There are concerns that the dosing interval in the two-dose regimen might be too long even in areas with low levels of SP resistance, and increasing resistance further shortens the duration of the post-treatment prophylactic effect [6], [12]. This concerns especially HIV-positive women not taking cotrimoxazole prophylaxis, who have increased susceptibility to malaria in pregnancy and reduced efficacy of antimalarial drugs [7], [8], [13]. Also, many pregnant women receive none or only one dose of SP despite making several antenatal care visits during the second and third trimesters [9]–[11]. One reason for this is the difficulty to determine the appropriate timing of the doses; monthly SP policy would simplify the dosing regimen and thus ensure that more women receive at least two doses of SP [10], [11], [15], [23], [24]. Previous results suggest that also monthly SP, alone and in combination with azithromycin, is safe to use during the second and third trimesters of pregnancy [13], [17], [25], [26]. All this, together with the ease of single-dose administration which can be done under direct observation at antenatal care visits, and the low price of SP, makes the monthly SP regimen seem suitable for IPTp until an effective, safe and feasible alternative for SP has been identified.

However, before adopting a monthly SP IPTp policy, several issues have to be taken into consideration. The prevalence of mutations in *P. falciparum* associated with resistance to SP has increased in Africa, and is very high among pregnant women in some parts of Malawi [4], [5]. There have been concerns that IPTp might increase resistance and even cause adverse outcomes, but the results have so far been contradictory [5], [27]–[31]. Additionally, local conditions such as malaria prevalence and the level of acquired malaria immunity among the target group need to be taken into consideration as they might affect SP IPTp efficacy. In our study area the level of resistance was high already during the enrollment between 2003 and 2007: over 80% of the malaria isolates collected among the LAIS participants at enrollment were found to express quintuple DNA mutations for SP resistance (Steven Meshnick et al, unpublished results).

In our previous analysis we found that the combination regimen of monthly SP and two doses of azithromycin, but not monthly SP alone, was significantly superior to the standard regimen of two SP doses in preventing preterm delivery and LBW [17]. Azithromycin is both a modestly active antimalarial and a potent broad-spectrum antimicrobial drug, and thus the improved efficacy of the combination could have been due to either activity [32], [33]. However, because the prevalence of microscopic malaria was low, we were earlier unable to adequately separate the antimalarial and other possible effects of azithromycin.

In the current analysis, we found that the monthly SP regimen was associated with statistically significant reductions in PCR-diagnosed malaria prevalence at delivery both alone, as well as in combination with azithromycin, and that there were no major differences between the two intervention regimens in the point-estimates for the effect size. The results thus suggest that the decrease in parasitaemia at delivery was mainly caused by the increased frequency of the SP dosage, while the improvement in birth outcomes might have been due to some other activity of azithromycin, independent of its activity against malaria. A possible explanation for azithromycin’s weak antimalarial effect in our study is the used dosage. Although 1g dose of azithromycin given twice several weeks apart is sufficient to cure many RTIs, it might be suboptimal against *P. falciparum* malaria [32], [33].

Two limitations of our study are that we analyzed the prevalence of malaria parasitaemia with PCR only at delivery, and only from peripheral blood. It could be argued that the difference in parasitaemia at delivery simply reflects the fact that the last dose in the regimens containing monthly SP was given nearer to delivery than in the two-dose regimen. Thus we cannot rule out that the groups might have had comparable malaria prevalence during pregnancy and that the differences only developed towards delivery. However, it is unlikely that the efficacy of SP would have differed during pregnancy, and therefore the monthly dosing with shorter treatment intervals is likely to have been more effective throughout pregnancy. This is supported by our finding of a comparable effect size in the prevalence of microscopic malaria parasitaemia at 32 gestational weeks in the original sample from which the current study population was drawn [17]. Another limitation is that we did not perform analyses with PCR of placental blood and did not collect histological samples of the placenta to diagnose placental malaria. The microscopic results of placental blood showed no difference between the groups [17].

In conclusion, our results suggest that increasing the frequency of SP administration during pregnancy improves efficacy against malaria at delivery among HIV-negative women as well as a population consisting of both HIV-positive and –negative pregnant women of all gravidities in a setting of relatively low but holoendemic malaria transmission, frequent use of bed nets and high SP resistance. However, further research is needed to study the consequences of the prevention of submicroscopic malaria infections to maternal and child health, as well as the effect of different levels of resistance and malaria prevalence on the IPTp efficacy. We therefore recommend the use of sensitive diagnostic tools for malaria, such as PCR and placental histology, and the determination of the level and change of drug resistance and acquired immunity in future IPTp trials.

## Supporting Information

Checklist S1 **CONSORT Checklist**
(DOC)

Protocol S1 **Trial Protocol**
(DOC)

## References

[1] Desai M, ter Kuile FO, Nosten F, McGready R, Asamoa K (2007). Epidemiology and burden of malaria in pregnancy.. Lancet Infect Dis.

[2] Steketee RW, Nahlen BL, Parise ME, Menéndez C (2001). The burden of malaria in pregnancy in malaria-endemic areas.. Am J Trop Med Hyg 64 (suppl).

[3] World Health Organization (WHO) (2011). World Malaria Report 2011.. Geneva: World Health Organization.

[4] Sridaran S, McClintock SK, Syphard LM, Herman KM, Barnwell J (2010). Anti-folate drug resistance in Africa: meta-analysis of reported dihydrofolate reductase (dhfr) and dihydropteroate synthase (dhps) mutant genotype frequencies in African Plasmodium falciparum parasite populations.. Malar J.

[5] Taylor SM, Antonia A, Feng G, Mwapasa V, Chaluluka E (2012). Adaptive evolution and fixation of drug-resistant Plasmodium falciparum genotypes in pregnancy-associated malaria: 9-year results from the QuEERPAM study.. Infect Genet Evol.

[6] White NJ (2005). Intermittent presumptive treatment for malaria.. PLoS Med.

[7] Menéndez C, D’Alessandro U, ter Kuile FO (2007). Reducing the burden of malaria in pregnancy by preventive strategies.. Lancet Infect Dis.

[8] Ter Kuile FO, Steketee RW (2007). Intermittent preventive therapy with sulfadoxine-pyrimethamine during pregnancy: seeking information on optimal dosing frequency.. J Infect Dis.

[9] van Eijk AM, Hill J, Alegana VA, Kirui V, Gething PW (2011). Coverage of malaria protection in pregnant women in sub-Saharan Africa: a synthesis and analysis of national survey data.. Lancet Infect Dis.

[10] Gill CJ, Macleod WB, Mwanakasale V, Chalwe V, Mwananyanda L (2007). Inferiority of single-dose sulfadoxine-pyrimethamine intermittent preventive therapy for malaria during pregnancy among HIV-positive Zambian women.. J Infect Dis.

[11] Sangaré LR, Stergachis A, Brentlinger PE, Richardson BA, Staedke SG (2010). Determinants of Use of Intermittent Preventive Treatment of Malaria in Pregnancy: Jinja, Uganda.. PLoS ONE.

[12] Diakite OS, Kayentao K, Traoré BT, Djimde A, Traoré B (2011). Superiority of 3 over 2 doses of intermittent preventive treatment with sulfadoxine-pyrimethamine for the prevention of malaria during pregnancy in Mali: a randomized controlled trial.. Clin Infect Dis.

[13] ter Kuile FO, van Eijk AM, Filler SJ (2007). Effect of sulfadoxine-pyrimethamine resistance on the efficacy of intermittent preventive therapy for malaria control during pregnancy: a systematic review.. JAMA.

[14] Parise ME, Ayisi JG, Nahlen BL, Schultz LJ, Roberts JM (1998). Efficacy of sulfadoxine-pyrimethamine for prevention of placental malaria in an area of Kenya with a high prevalence of malaria and human immunodeficiency virus infection.. Am J Trop Med Hyg.

[15] Filler SJ, Kazembe P, Thigpen M, Macheso A, Parise ME (2006). Randomized trial of 2-dose versus monthly sulfadoxine-pyrimethamine intermittent preventive treatment for malaria in HIV-positive and HIV-negative pregnant women in Malawi.. J Infect Dis.

[16] Hamer DH, Mwanakasale V, Macleod WB, Chalwe V, Mukwamataba D (2007). Two-dose versus monthly intermittent preventive treatment of malaria with sulfadoxine-pyrimethamine in HIV-seropositive pregnant Zambian women.. J Infect Dis.

[17] Luntamo M, Kulmala T, Mbewe B, Cheung YB, Maleta K (2010). Effect of repeated treatment of pregnant women with sulfadoxine-pyrimethamine and azithromycin on preterm delivery in Malawi: a randomized controlled trial.. Am J Trop Med Hyg.

[18] Rantala AM, Taylor SM, Trottman PA, Luntamo M, Mbewe B (2010). Comparison of real-time PCR and microscopy for malaria parasite detection in Malawian pregnant women.. Malar J.

[19] Kulmala T, Vaahtera M, Ndekha M, Koivisto AM, Cullinan T (2001). Gestational health and predictors of newborn weight amongst pregnant women in rural Malawi.. Afr J Reprod Health.

[20] Mayor A, Serra-Casas E, Bardají A, Sanz S, Puyol L (2009). Sub-microscopic infections and long-term recrudescence of Plasmodium falciparum in Mozambican pregnant women.. Malar J.

[21] Mockenhaupt FP, Rong B, Till H, Eggelte TA, Beck S (2000). Submicroscopic Plasmodium falciparum infections in pregnancy in Ghana.. Trop Med Int Health.

[22] Adegnika AA, Verweij JJ, Agnandji ST, Chai SK, Breitling LP (2006). Microscopic and sub-microscopic Plasmodium falciparum infection, but not inflammation caused by infection, is associated with low birth weight.. Am J Trop Med Hyg.

[23] Ouma PO, Van Eijk AM, Hamel MJ, Sikuku E, Odhiambo F (2007). The effect of health care worker training on the use of intermittent preventive treatment for malaria in pregnancy in rural western Kenya.. Trop Med Int Health.

[24] Ashwood-Smith H, Coombes Y, Kaimila N, Bokosi M, Lungu K (2002). Availability and use of sulphadoxine–pyrimethamine (SP) in pregnancy in Blantyre District: a safe motherhood and Blantyre integrated malaria initiative (BIMI) joint survey.. Malawi Medical Journal.

[25] Peters PJ, Thigpen MC, Parise ME, Newman RD (2007). Safety and toxicity of sulfadoxine/pyrimethamine: implications for malaria prevention in pregnancy using intermittent preventive treatment.. Drug Saf.

[26] Kalilani L, Mofolo I, Chaponda M, Rogerson SJ, Alker AP (2007). A randomized controlled pilot trial of azithromycin or artesunate added to sulfadoxine-pyrimethamine as treatment for malaria in pregnant women.. PLoS One.

[27] Harrington WE, Mutabingwa TK, Muehlenbachs A, Sorensen B, Bolla MC (2009). Competitive facilitation of drug-resistant Plasmodium falciparum malaria parasites in pregnant women who receive preventive treatment.. Proc Natl Acad Sci U S A.

[28] Harrington WE, Mutabingwa TK, Kabyemela E, Fried M, Duffy PE (2011). Intermittent treatment to prevent pregnancy malaria does not confer benefit in an area of widespread drug resistance.. Clin Infect Dis.

[29] Mockenhaupt FP, Bedu-Addo G, Eggelte TA, Hommerich L, Holmberg V (2008). Rapid increase in the prevalence of sulfadoxine-pyrimethamine resistance among Plasmodium falciparum isolated from pregnant women in Ghana.. J Infect Dis.

[30] Bertin G, Briand V, Bonaventure D, Carrieu A, Massougbodji A (2011). Molecular markers of resistance to sulphadoxine-pyrimethamine during intermittent preventive treatment of pregnant women in Benin.. Malar J.

[31] Taylor SM, Antonia AL, Chaluluka E, Mwapasa V, Feng G (2012). Antenatal Receipt of Sulfadoxine-Pyrimethamine Does Not Exacerbate Pregnancy-Associated Malaria Despite the Expansion of Drug-Resistant Plasmodium falciparum: Clinical Outcomes From the QuEERPAM Study.. Clin Infect Dis.

[32] Chico RM, Pittrof R, Greenwood B, Chandramohan D (2008). Azithromycin-chloroquine and the intermittent preventive treatment of malaria in pregnancy.. Malar J.

[33] Pfizer Inc. Zithromax® product information.. http://labeling.pfizer.com/ShowLabeling.aspx?id=511.

